# Supplementary figures and images for: African eggplant-associated virus: Characterization of a novel tobamovirus identified from Solanum macrocarpon and assessment of its potential impact on tomato and pepper crops
Source: PLoS One. 2023 Apr 13;18(4):e0277840. doi: 10.1371/journal.pone.0277840 (PMC10101479; doi:10.1371/journal.pone.0277840)

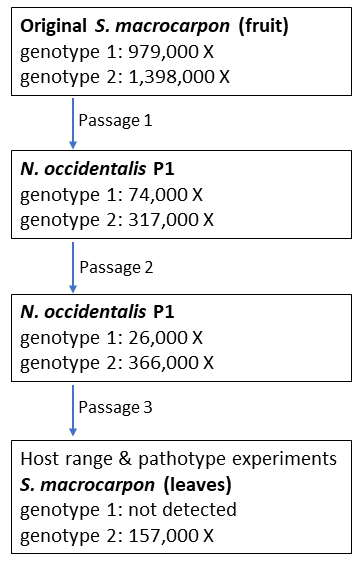

Supplement: S1 Fig — (TIF) [file pone.0277840.s001.tif]

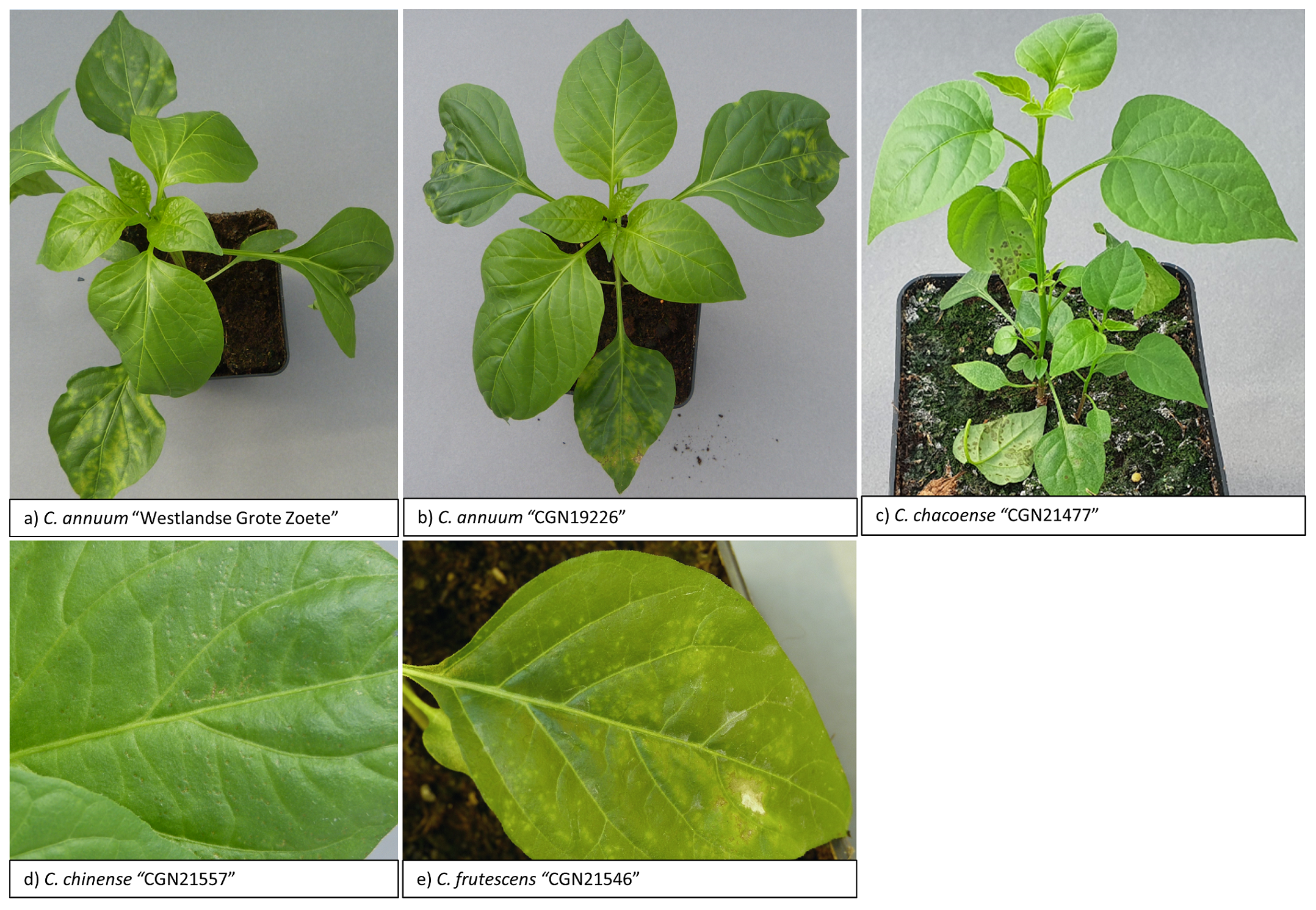

Supplement: S2 Fig — a) Capsicum annuum “Westlandse Grote Zoete”: chlorotic lesions, b) C. annuum “CGN19226”: chlorotic lesions, leaf bulging, c) C. chacoense “CGN21477”: necrotic lesions, leaf abscission, d) C. chinense “CGN21557”: necrotic lesions, e) C. frutescens “CGN21546”: chlorotic and necrotic lesions. (TIF) [file pone.0277840.s002.tif]
