# Supplementary material for: African eggplant-associated virus: Characterization of a novel tobamovirus identified from Solanum macrocarpon and assessment of its potential impact on tomato and pepper crops
Source: PLoS One. 2023 Apr 13;18(4):e0277840. doi: 10.1371/journal.pone.0277840 (PMC10101479; doi:10.1371/journal.pone.0277840)
Supplement: Attachment — Submitted filename: Response to Reviewers.docx [file pone.0277840.s004.docx]

Dear editor, dear reviewer,

Thank you for the time and effort that you dedicated to provide useful feedback on our manuscript. Please find our response to each point raised by reviewer #1 below:

- Reviewer: In this study, the authors aimed to identify viruses infecting Solanum species and their potential hosts. As a result of RNA sequencing and bioinformatic analyses of imported eggplant fruit from Mexico, a novel virus was identified and named African eggplant-associated virus. This manuscript is valuable for identifying the novel virus and providing information on its potential hosts and resistant cultivars.

Authors: Thank you for the appreciation of our work.

- Reviewer: The introduction section should focus on known viruses infecting Solanum species and background information on this study.

Authors: A very wide range of viruses are known to infect *Solanum* species, too many to describe in the introduction. The introduction focuses on the *Tobamovirus* genus, because the novel virus belongs to this group.

- Reviewer: At the end of the introduction, the purpose of this study, which does not include any results from this study, can be briefly described.

Authors: We rephrased the last paragraph of the introduction to clarify the purpose of the study.

- Reviewer: The manuscript is well-written, and the abstract is nicely written.

Authors: Thank you for your appreciation.

- Reviewer: The results from this study are presented in Figure 1, which could be moved to the Results section.

Authors: Figure 1 has been moved to the Results section.

- Reviewer: It is suggested to provide the abbreviation for "The International Plant Protection Convention," and the materials and methods should describe the details on mechanical passages and reagents/equipment's used in this study.

Authors: The abbreviation “IPPC” has been added to line 35. The Materials and Methods section has been slightly restructured, to clarify that the procedure and reagents/equipment of the mechanical passages have been described in Verhoeven and Roenhorst (2000) to which is referred.

- Reviewer: Maximum likelihood method should be used instead of Neighbor-Joining method, and the abbreviation for the virus can be described in Line 168. Additionally, it would be helpful to generate a new phylogenetic tree for Figure 2 with Maximum likelihood method and to show the genome organization of the novel virus.

Authors: We also generated a phylogenetic tree using RAxML (Randomized Axelerated Maximum Likelihood) in Geneious. This has been added to Materials & Methods line 134-135. The positioning of the novel virus within the tree, and the positioning of the other viruses relative to each other, was the same as when the Neighbor-Joining method was used, which has been added to line 177-179. Therefore, Figure 2 presents a reliable phylogenetic tree.

The genome organization of the novel virus corresponds to the genome organization of other tobamoviruses, with four open readings frames (ORFs), encoding a small replicase subunit, an RNA-dependent RNA polymerase (translated through ribosomal readthrough at the stop codon of the small replicase subunit), a movement protein, and a coat protein (as described on line 164-167).

The abbreviation for the virus (AEaV) has been added to line 175 (previously line 168), after which the abbreviation is used.

- Reviewer: The tables show excellent results, and overall, this manuscript is well-done. Congratulations!

Authors: We highly appreciate your feedback and have done our best to improve the manuscript considering your suggestions.

Yours sincerely,

Anne Giesbers, PhD
